# Continuous Non-Invasive Monitoring of Tidal Volumes by Measurement of Tidal Impedance in Neonatal Piglets

**DOI:** 10.1371/journal.pone.0021003

**Published:** 2011-06-07

**Authors:** Florian Kurth, Fabienne Zinnow, Alexandra Prakapenia, Sabrina Dietl, Stefan Winkler, Sascha Ifflaender, Mario Rüdiger, Wolfram Burkhardt

**Affiliations:** Department of Neonatology and Paediatric Intensive Care, University Hospital Carl Gustav Carus, Dresden, Germany; Erasmus University Rotterdam, Netherlands

## Abstract

**Background:**

Electrical Impedance measurements can be used to estimate the content of intra-thoracic air and thereby give information on pulmonary ventilation. Conventional Impedance measurements mainly indicate relative changes, but no information concerning air-volume is given. The study was performed to test whether a 3-point-calibration with known tidal volumes (VT) during conventional mechanical ventilation (CMV) allows subsequent calculation of VT from total Tidal-Impedance (tTI) measurements using Quadrant Impedance Measurement (QIM). In addition the distribution of TI in different regions of the thorax was examined.

**Methodology and Principal Findings:**

QIM was performed in five neonatal piglets during volume-controlled CMV. tTI values at three different VT (4, 6, 8 ml/kg) were used to establish individual calibration curves. Subsequently, each animal was ventilated with different patterns of varying VT (2–10 ml/kg) at different PEEP levels (0, 3, 6, 9, 12 cmH_2_O). VT variation was repeated after surfactant depletion by bronchoalveolar lavage. VT was calculated from tTI values (VT_calc_) and compared to the VT delivered by the ventilator (VT_PNT_). Bland-Altman analysis revealed good agreement between VT_calc_ and VT_PNT_ before (bias −0.08 ml; limits of agreement −1.18 to 1.02 ml at PEEP = 3 cmH_2_O) and after surfactant depletion (bias −0.17 ml; limits of agreement −1.57 to 1.22 ml at PEEP = 3 cmH_2_O). At higher PEEP levels VT_calc_ was lower than VT_PNT_, when only one fixed calibration curve (at PEEP 3 cmH_2_O) was used. With a new calibration curve at each PEEP level the method showed similar accuracy at each PEEP level. TI showed a homogeneous distribution over the four assessed quadrants with a shift toward caudal regions of the thorax with increasing VT.

**Conclusion:**

Tidal Impedance values could be used for precise and accurate calculation of VT during CMV in this animal study, when calibrated at each PEEP level.

## Introduction

Measurements of lung volume changes are a prerequisite to optimize mechanical respiratory support and study respiratory mechanics. In order to avoid interference with the patient and the respiratory support, these measurements should ideally be non invasive and should not increase breathing load or dead space. In addition to global data on tidal volume (VT) and functional residual capacity (FRC), information on regional air distribution would be of interest. In neonates the restrictions of the currently available methods, such as interference with spontaneous breathing, limit the possibility of reliable and practical bedside monitoring of lung volumes in clinical routine [1], [2].

Electrical Impedance measurement is a non-invasive, radiation-free bedside technique which determines changes in electrical conductivity of organs based on the variability in electrical impedance between tissue, air and fluid. The technology has been proposed for the estimation of lung volume changes more than 20 years ago [3] and has been widely employed for surveillance of the respiratory frequency in the form of impedance pneumography in patient monitors for a long time [4]. Moreover, impedance measurements can be used to generate cross-sectional images of impedance distribution within the thorax, a technique which is known as electrical impedance tomography (EIT) and which has gained heightened interest in respiratory and intensive care recently [5].

Whereas EIT produces colour coded information on relative impedance changes, it does not allow quantification of impedance changes of the lung and expression as absolute volumes. Quadrant Impedance Measurement (QIM) uses a different approach of impedance measurement which virtually divides the thorax in 4 quadrants and determines impedance changes in these 4 regions. The amplitudes of impedance changes during respiratory cycles are thereby expressed as Tidal Impedance (TI).

The present animal study was performed to answer the following questions: 1. How precisely and how accurately can VT in millilitres be estimated by total Tidal Impedance (tTI i.e. the sum of TI-values from all four quadrants) after three-point calibration with known tidal volumes during conventional mechanical ventilation? 2. Can one single calibration procedure (at PEEP 3 cmH_2_O) be used for different PEEP levels or should individual calibrations be performed? 3. How is the precision and accuracy of the method influenced by lung injury as implemented by saline lavage?

## Materials and Methods

### Animals

The study was performed in five anaesthetized newborn piglets and all experiments were approved by the university and the state committee for animal care and adhered to the national law on the care and use of laboratory animals. Animals were treated as previously described [6]. In short, Ketamin (20 mg/kg) and Azaperon (5 mg/kg) were injected intramuscularly for pre-medication. Anaesthesia was induced by intravenous administration of Morphine (2 mg/kg) and Midazolam (2 mg/kg) and subsequently maintained with Morphine (20 µg/kg/hr), Midazolam (2 mg/kg/hr) and Rocuronium (2 mg/kg/hr). An arterial line was placed in the femoral artery, connected with a pressure transducer and kept open with a continuous infusion of heparin-saline mixture (1 ml/hr). Animals were intubated via tracheotomy (tubes of 3.5–4.5 mm inner diameter with a side port, Vygon, Ecouen, France) and ventilated in supine body position in a volume-controlled mode of ventilation (Stephanie, Fa. Stephan, Gackenbach, Germany). Initial ventilator settings were as follows: respiratory rate 60 breaths/min, inspiratory time 0.4 sec, fractional concentration of oxygen in inspired gas 1.0, tidal volume 6 ml/kg, positive end-expiratory pressure (PEEP) 4 cmH_2_O. Monitoring of vital parameters was performed using a neonatal patient monitor (HP, Palo Alto, CA, USA). Blood gas analysis was performed once at every PEEP level (ABL 500, Radiometer, Copenhagen, Denmark). Surfactant depletion was implemented by repetitive bronchoalveolar with warmed saline (30 ml/kg) as originally described by Lachmann *et al.* [7]. The lavage procedure was repeated until PaO_2_ remained below 13 kPa for at least 30 minutes.

### Experimental Protocol

Stepwise variation of five different VT (2, 4, 6, 8, 10 ml/kg) was performed in 3 different patterns: 1. Increasing/decreasing (2-4-6-8-10-8-6-4-2 ml/kg), 2. Antipodal around VT 6 ml/kg (6-2-6-10-6-4-6-8-6), 3. Random (using every one of the above indicated VT values twice in random order). VT was kept constant for 30 seconds at each step of variation. The identical variation was performed at 5 different PEEP levels (0, 3, 6, 9, 12 cmH2O), except for the random part which was different at every PEEP level.

VT-variation was performed before and after surfactant depletion following the identical protocol, resulting in 280 per animal in total.

Measurements of VT were performed at the Y piece of the ventilator using the fixed orifice pneumotachograph (PNT) of the Stephanie ventilator (resistance 1.1 kPa/L/s at 5 L/min flow, dead space 0.9 mL). The individual differential-pressure/flow characteristic curve of the pneumotachograph was determined prior to the experiments (Schaller Medizintechnik, Dresden, Germany) in order to obtain the coefficients for a fourth grade polynomial model for calculation of VT by the ventilator. Calibration of the PNT with the built-in test-lung was carried out according to the manufacturer's instruction prior to each experiment [8]–[10].

### Impedance Measurements

**Figure 1:**
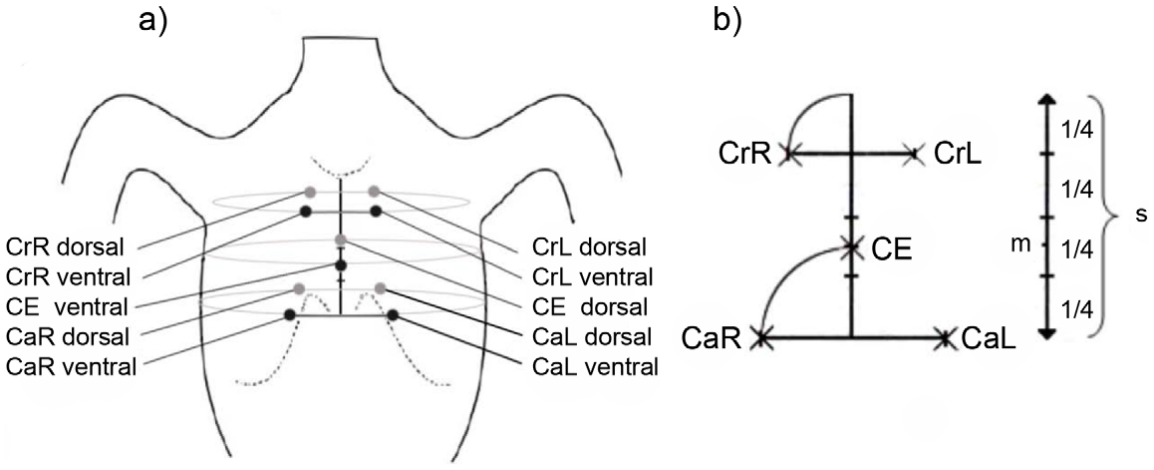
Placement of electrodes. a) Schematic map of the piglet's body and position of electrodes; b) detailed illustration of the sternum and distances of the electrodes; the length of the sternum (s) is quartered. Cranial Right (CrR) and Cranial Left (CrL) ventral are set at the end of the first quarter from the cranial end of the sternum right and left with the distance ¼ s from the axis. The central electrode for current injection (CE) is set in the middle (m) of the remaining ¾ of the sternum. Caudal Right (CaR) and Caudal Left (CaL) are placed right and left from the caudal end of the sternum with the distance between m and the caudal end of the sternum. Dorsal electrodes are set mirror-inverted to the ventral electrodes.

Impedance measurements were performed using the EIS/Quadrant Impedance Monitoring (QIM device of EMS Biomedical (EMS, Korneuburg, Austria). The device measures Tidal Impedance (TI) from the amplitudes of thoracic impedance changes during a ventilatory cycle. A constant current source which generates a sinus-wave output current of 400 µA at a frequency of 5 KHz was used and current was applied via one ventral and one dorsal current electrode. Voltage measurements for subsequent determination of impedance were performed in four regions of the thorax, cephalic left, cephalic right and caudal left and right at a frequency of 500 Hz. For each region one ventral and one dorsal electrode was placed according to a standardized placement procedure (Figure 1). Disposable adhesive EEG electrodes were used for both current injection and voltage measurements (Neuroline 720, Ambu, Bad Nauheim, Germany). Tidal Impedance in the four quadrants was recorded separately via four channels and expressed as quadrant Tidal Impedance (qTI) in Arbitrary Units (AU). Total Tidal Impedance (tTI) was subsequently calculated as sum of qTI from all four channels. A Bandpass filter with a lower frequency of 0.2 Hz and an upper frequency of 1.5 Hz was used to process incoming biosignals of the four channels. Data were transferred to a PC via USB interface and analyzed with EIM client 4.0 software (EMS, Korneuburg, Austria).

### Analysis of variability of TI

To investigate the within-subject variability of impedance measurements during subsequent breathing cycles tTI values of one random sample of 10 breathing cycles at a constant VT = 6 ml/kg at PEEP = 3 cmH_2_O were assessed for each animal and compared to the variability of volumes as measured by the PNT at a constant VT = 6 ml/kg. For subsequent calculation of tTI the mean amplitude of 5 breathing cycles was used once VT had stabilized at the respective step of variation.

In a second step, variability of impedance measurements during repeated measurements was assessed using all tTI values of corresponding VT levels during VT variation at one PEEP level.

### Calibration procedure

Three point calibration of tTI was performed prior to VT variation at each PEEP level in every animal, using three pre-specified VT levels (4, 6, 8 ml/kg). Linear regression analysis was performed in order to compute an equation which allowed calculation of VT (VT_calc_) from tTI based on the three tTI measurements from known VT during calibration (VT_calc_ = intercept+slope×tTI).

VT was calculated in two different ways: 1.using one calibration at PEEP 3 cmH_2_O for all VT at all 5 PEEP levels. 2. Using the individual calibration from each PEEP level for calculation of VT at the respective PEEP level. Bland-Altman analysis was performed in order to compare VT_calc_ with VT as indicated from the PNT (VT_PNT_) [11].

### Analysis of distribution of TI in different quadrants

Analysis of distribution of TI was performed based on the separate data from the 4 different quadrants (qTI). Therefore the fraction of qTI divided by tTI was computed for all TI measurements and expressed as fractional TI (fTI) for every quadrant. Changes of fTI were assessed for different VT at different PEEP levels.

### Statistical analysis

Data of Impedance Measurements was exported to MS excel and JMP (JMP 7.0, SAS Institute Inc., NC, USA) via EIM client 4.0 software data export. Bland Altman analysis was performed using a plug-in for MS excel (analyze-it.com). Bias and Limits of agreement were calculated from the means of corresponding VT from individual piglets. Variability of tTI was expressed as coefficient of variation (CV). Data are expressed as mean (±SD) if normal distribution was assumed or median (range) in case no normal distribution was assumed.

## Results

**Figure 2:**
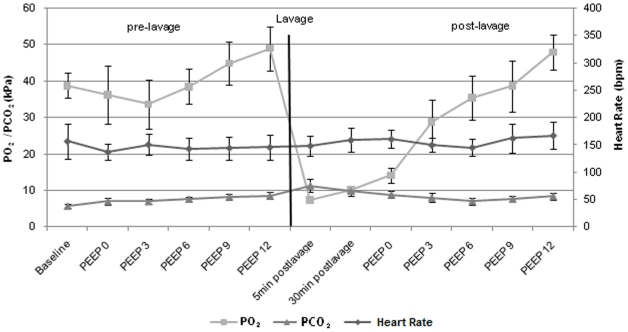
Blood Gases and Heart Rate. Shown are mean (SD) values of blood gases and heart rate for n = 5 animals. Blood gas analyses were performed after measurements at one PEEP level before changing to the next PEEP level.

**Figure 3:**
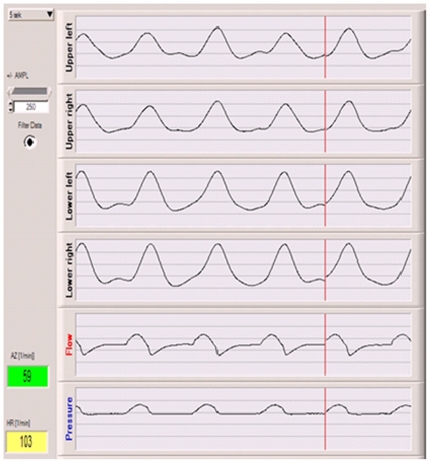
Tracing signal of impedance measurement during 5 breathing cycles. Shown are tracing signals of QIM measurements from 4 quadrants during 5 respiratory cycles. In addition flow and pressure curves from the ventilator are shown below in order to illustrate the time course of impedance changes with respect to ventilatory cycles from the ventilator.

Animals had a median (range) age of 8 (1–14) days and a bodyweight of 1.9 (1.6–3.0) kg. All five animals remained stable during the experimental period and no additional interventions were necessary. A median (range) of 4 (3–7) bronchoalveolar lavages were required to achieve stable post-lavage PaO_2_ levels below 13 kPa. Figure 2 depicts the courses of blood gases and heart rate during the experiments. Continuous recording of impedance measurements was possible in all animals and TI measurements were available for all 280 VT variations in all animals resulting in 1400 measurements in total. Figure 3 shows a representative tracing signal from impedance measurements during 5 breathing cycles.

### Within-subject variability of impedance measurements

Assessment of tTI values from 10 consecutive breathing cycles at VT = 6 ml/kg in each animal showed median (range) CV of 0.08 (0.04–0.10) and 0.07 (0.03–0.07) pre- and post-lavage, respectively. This variability was comparable to the variability of VT between single breathing cycles as delivered by the ventilator and measured by the pneumotachograph (0.06, range 0.05–0.09 and 0.06, range 0.04–0.09, pre- and post-lavage, respectively).

Median within-subject variability of repeated tTI measurements of corresponding VT levels at different time points during variation at one PEEP level ranged from 0.10 (0.04–0.19) and 0.08 (0.02–0.14) for VT = 2 ml/kg to 0.03 (0.01–0.06) and 0.03 (0.01–0.08) for VT = 10 ml/kg for pre- and post-lavage, respectively (Table 1).

**Table 1:** Within subject variability of repeated tTI measurements.

	Prelavage					Postlavage				
Vt (ml/kg)	2	4	6	8	10	2	4	6	8	10
**PEEP 0**	0,10(0,04–0,19)	0,08(0,05–0,10)	0,06(0,05–0,06)	0,04(0,02–0,08)	0,03(0,01–0,04)	0,08(0,02–0,14)	0,04(0,02–0,07)	0,05(0,03–0,08)	0,03(0,02–0,04)	0,02(0,02–0,03)
**PEEP 3**	0,08(0,05–0,15)	0,07(0,01–0,10)	0,05(0,03–0,06)	0,03(0,02–0,04)	0,01(0,01–0,06)	0,08(0,07–0,22)	0,05(0,04–0,08)	0,04(0,03–0,10)	0,03(0,02–0,09)	0,03(0,02–0,06)
**PEEP 6**	0,09(0,03–0,22)	0,04(0,03–0,09)	0,04(0,03–0,09)	0,03(0,03–0,05)	0,02(0,01–0,03)	0,06(0,03–0,11)	0,05(0,03–0,15)	0,04(0,03–0,07)	0,02(0,02–0,06)	0,02(0,02–0,03)
**PEEP 9**	0,07(0,04–0,37)	0,07(0,03–0,09)	0,05(0,02–0,08)	0,04(0,02–0,04)	0,03(0,01–0,04)	0,11(0,07–0,18)	0,050,03–0,13)	0,05(0,03–0,10)	0,03(0,02–0,07)	0,03(0,01–0,08)
**PEEP 12**	0,10(0,04–0,13)	0,06(0,02–0,10)	0,04(0,03–0,06)	0,03(0,02–0,06)	0,02(0,01–0,04)	0,08(0,03–0,20)	0,04(0,02–0,07)	0,05(0,04–0,06)	0,02(0,01–0,08)	0,02(0,02–0,03)

Shown are median (range) coefficients of variation (CV) of repeated tTI measurements for corresponding VT levels at different PEEP levels Pre- and Post-lavage for n = 5 animals. Repeated measurements are taken at different time points during variation at one PEEP level.

### Derivation of Calibration Equations

A strong linear relationship was found between tTI and VT in all animals at all PEEP levels for pre-lavage and post-lavage measurements (all R^2^>0.96). Visual examination of scatter plots showed homoscedasticity and normal distribution of residuals. No statistically significant relationship was found between intercepts or slopes of calibration curves and the animals' body weights or the PEEP levels. VT-estimation was first performed based on intercepts and slopes from calibration at PEEP 3 cmH_2_O and second from calibration at the respective PEEP level.

### VT-estimation based on calibration at PEEP 3 cmH_2_O

When VT_calc_ was calculated based on three point calibration at PEEP = 3 cmH_2_O there was a good agreement between VT_calc_ and VT_PNT_ at PEEP = 3 cmH_2_O for pre- and post-lavage measurements (bias −0.08 ml and −0.17 ml, limits of agreement −1.18 to 1.02 ml and −1.57 to 1.22 ml, respectively) (Table 2). At higher PEEP levels VT_calc_ based on calibration at PEEP 3 cmH_2_O slightly underestimated VT_PNT_ (bias −1.60 ml; limits of agreement −4.75 to 1.56 ml at PEEP = 12 cmH_2_O with calibration at PEEP = 3 cmH_2_O pre-lavage). This difference was even more marked for post-lavage measurements (bias −3.04 ml; limits of agreement −8.37 to 2.29 ml at PEEP = 12 cmH_2_O with calibration at PEEP = 3 cmH_2_O post-lavage). The results were not affected by the different patterns of VT variation (data not shown).

**Table 2:** Agreement between VT_calc_ and VT_PNT_ in Bland-Altman analysis.

		Pre-lavage		Post-lavage	
		Calibration at PEEP = 3 cmH_2_O	Calibration at same PEEP level	Calibration at PEEP = 3 cmH_2_O	Calibration at same PEEP level
PEEP 0 cmH_2_0	Bias (ml)	−0.25	0.42	0.11	0.37
	Limits of agreement (ml)	−2.05–1.40	0.71–1.56	−2.04–2.26	−1.24–1.98
PEEP 3 cmH_2_0	Bias (ml)	−0.08		−0.17	
	Limits of agreement (ml)	−1.18–1.02		−1.57–1.22	
PEEP 6 cmH_2_0	Bias (ml)	−0.50	−0.21	−1.29	−0.23
	Limits of agreement (ml)	−3.35–2.34	−2.34–1.92	−3.86–1.27	−1.49–1.02
PEEP 9 cmH_2_0	Bias (ml)	−1.00	−0.52	−2.29	−0.41
	Limits of agreement (ml)	−4.66–2.66	−2.75–1.72	−6.35–1.78	−1.53–0.07
PEEP 12 cmH_2_0	Bias (ml)	−1.60	−0.48	−3.04	−0.56
	Limits of agreement (ml)	−4.75–1.56	−2.61–1.65	−8.37–2.29	−2.20–1.08

### VT-estimation based on individual calibrations at respective PEEP levels

**Figure 4:**
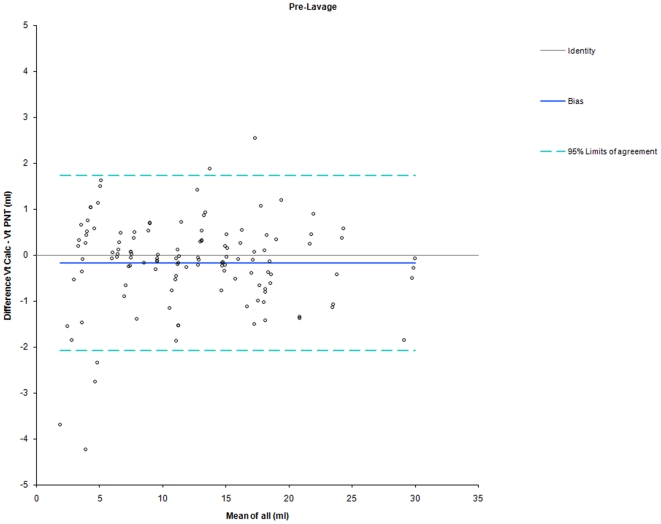
Bland-Altman Plot for comparison between VT_calc_ and VT_PNT_ during pre-lavage measurements. Shown are Line of Identity (grey line), Bias (blue line), and 95%-Limits of Agreement (dashed line). Calibration was performed at each PEEP level in all animals. Data points indicate the means of measurements in individual piglets at different PEEP levels.

**Figure 5:**
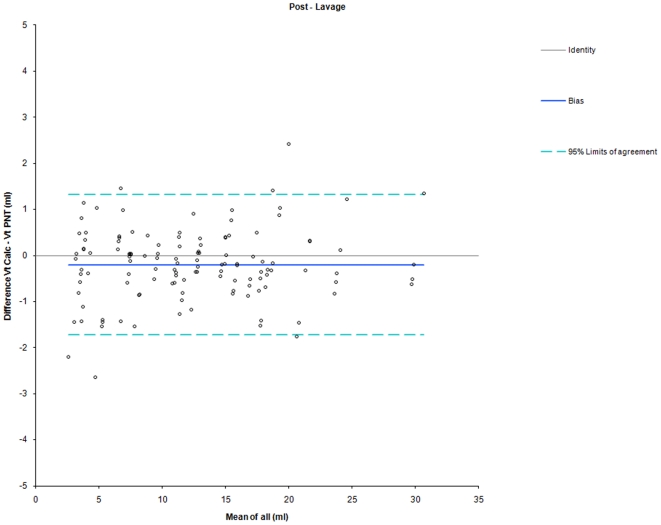
Bland-Altman Plot for comparison between VT_calc_ and VT_PNT_ during post-lavage measurements. Shown are Line of Identity (grey line), Bias (blue line), and 95%-Limits of Agreement (dashed line). Calibration was performed at each PEEP level in all animals. Data points indicate the means of measurements in individual piglets at different PEEP levels.

When VT_calc_ was calculated based on individual three-point calibrations obtained at the respective PEEP levels the difference between VT_calc_ and VT_PNT_ was markedly reduced compared to estimation based on one calibration at PEEP = 3 cmH_2_O (bias −0.48 ml and −0.56 ml, limits of agreement −2.61 to 1.65 ml and −2.20 to 1.08 ml at PEEP = 12 cmH_2_O with calibration at PEEP = 12 cmH_2_O). There was a similar accuracy and precision between pre- and post-lavage measurements (Table 2, Figures 4 and 5).

### Distribution of TI in single quadrants

Analysis of fTI in the single quadrants revealed a relatively homogeneous distribution of TI for pre- and post-lavage measurements. Both cranial quadrants exhibited mean fTI values of 0.22±0.03 pre-lavage, whereas the caudal quadrants showed mean fTI values of 0.32±0.03 and 0.24±0.03 for CaR and CaL, respectively. During post-lavage measurements, mean fTI values were 0.23±0.03 and 0.21±0.03 for CrR and CrL and 0.31±0.03 and 0.25±0.03 for CaR and CaL, respectively.

**Figure 6:**
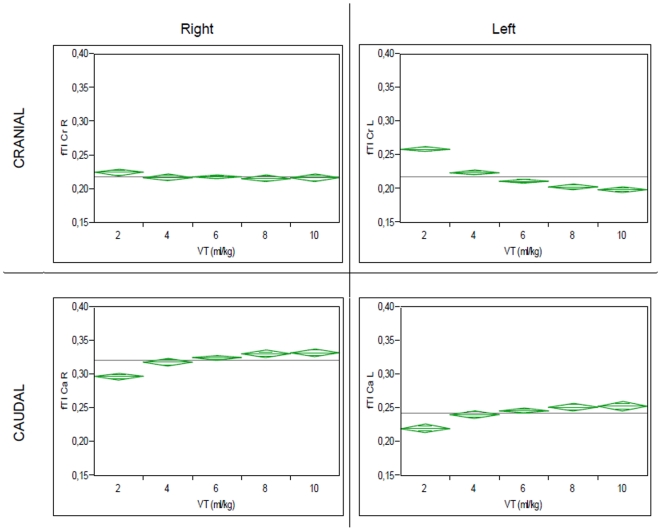
Distribution of fTI in different quadrants at different VT, pre-lavage. Shown are mean (grey line) fTI values for different VT, with 95%-Confidence Interval of the mean (green diamonds).

**Figure 7:**
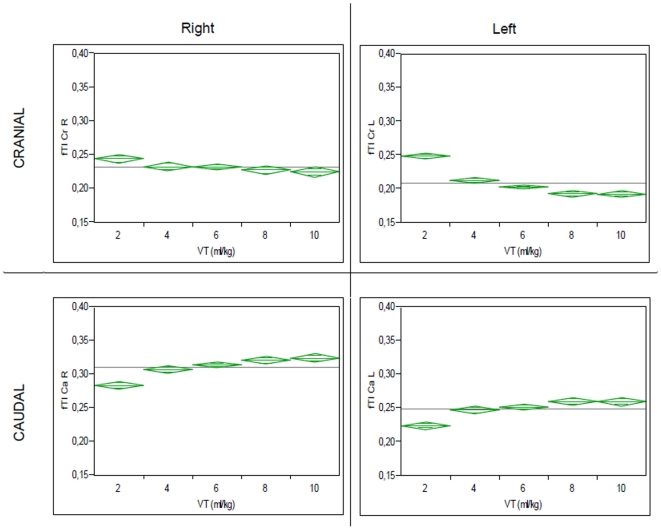
Distribution of fTI in different quadrants at different VT, post-lavage. Shown are mean (grey line) fTI values for different VT, with 95%-Confidence Interval of the mean (green diamonds).

As shown in Figure 6, analysis of fTI at different VT showed a small but consistent decrease of fTI with increasing VT in the cranial quadrants (0.22±0.04 and 26±0.04 for Vt 2 ml/kg versus 0.22±0.02 and 0.20±0.02 for Vt 10 ml/kg for CrR and CrL, respectively; pre-lavage data) and increasing fTI in the caudal quadrants (0.30±0.04 and 0.22±0.03 for Vt 2 ml/kg versus 0.33±0.02 and 0.25±0.03 for Vt 10 ml/kg for CaR and CaL, respectively;). This shift of fTI from cranial to caudal quadrants with increasing VT was equally observed for post-lavage measurements (Figure 7).

**Figure 8:**
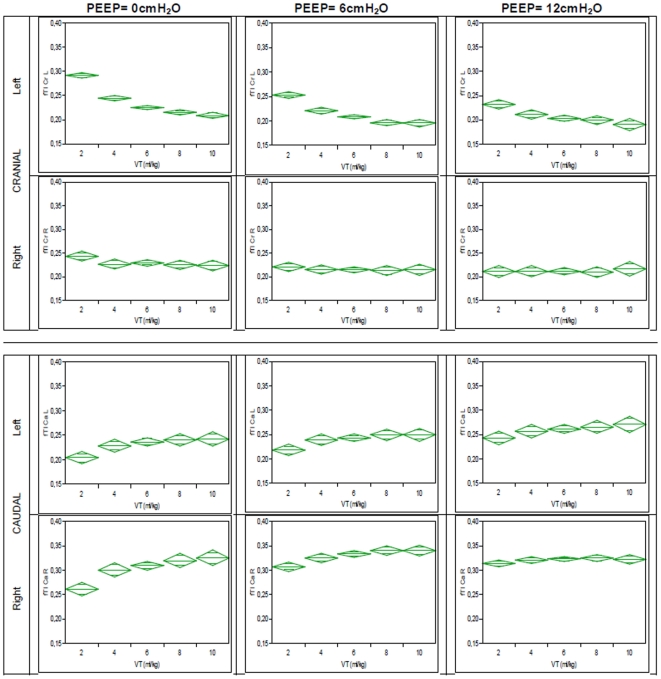
Distribution of fTI at different VT stratified according to PEEP, pre-lavage. Shown are mean fTI values (grey line) for different VT at different PEEP with 95%-Confidence Interval of the mean (green diamonds).

The cranio-caudal shift of fTI was more marked at lower PEEP levels than at higher PEEP levels (Figure 8).

## Discussion

The present study aimed to investigate whether measurements of impedance changes during ventilatory cycles could be used for non-invasive monitoring of VT in neonatal piglets. Impedance measurements were stable and reproducible during the study period as indicated by small within-subject variability. The exhibited limits of agreement and levels of bias between VT_calc_ and VT_PNT_ after three-point calibration at individual PEEP levels showed that accurate and precise calculation of VT from tTI values was possible. Moreover information on the distribution of TI was obtained for different VT.

The VT used for the calibration procedure are within the range of physiological breathing cycles of neonates (4–8 ml/kg) and could probably be used for calibration in a clinical setting without harm for the patient.

A tendency towards lower VT_calc_ was observed at higher pressure levels compared to lower pressure levels. This slight non-linearity between impedance change and lung volume change could have several reasons:

Although electrical impedance change of the thorax is mainly influenced by the amount of air inspired, other factors such as change of blood volume in the lung or occurrence of pulmonary oedema also have an impact on the resistivity change of the lung tissue [12], [13]. As heart rate and arterial pressure remained relatively stable in all animals throughout the experiments, a non-linear relationship between airway pressure and blood volume within the lung is not very likely, although it cannot be completely ruled out with the experimental setting of this study. The emergence of pulmonary oedema, however, was not examined in our study and could possibly have impaired electrical impedance in the course of the experiments, especially after induction of lung damage by bronchoalveolar lavage. The stability of measurements within the course of variation on one PEEP level however contradicts a strong influence of emergence of oedema.

The increasing compression of air could be another possible reason for comparatively smaller impedance changes at higher pressure levels. Assuming an atmospheric pressure of 1013 mbar, an increase of PEEP from 0 to 12 cmH_2_O results in a 1.2% increased compression of air. As mean airway pressure rises non-linearly with decreasing compliance at higher pressure levels, this compression could possibly even account for up to 2% loss of volume at higher pressure levels.

Redistribution of air from alveolar regions to the trachea and upper airways is another possible factor for the observed non-linearity between tTI and VT at higher pressure levels. Due to the position of electrodes impedance changes in this study presumably reflect volume changes in the lung periphery more than in the trachea and in central airways. Shift of ventilation from peripheral to central airways, possibly due to alveolar over-distension at higher pressure levels, would therefore result in comparatively smaller VT_calc_ as observed in our experiments. As this shift from peripheral regions to central airways is mainly determined by the relation between alveolar and tracheal/central airway compliance, it would further increase with decreasing alveolar compliance. This could be one possible explanation for the even more marked tendency towards lower VT_calc_ after induction of lung injury, when compliance of alveolar regions declines relatively to tracheal compliance.

A relatively homogeneous distribution of TI in the single quadrants was observed in this study. Whereas both cranial quadrants (CrR and CrL) exhibited similar fTI values, a higher fTI was observed in the CaR quadrant compared to CaL. This finding reflects most likely the comparatively reduced ventilation of the left lung caused by the anatomical differences due to the location of the mediastinum. Interestingly, a small but consistent shift of fTI from cranial to caudal quadrants was observed with increasing VT (Figures 6 and 7). Recruitment of caudal regions of the lung at larger VT, possibly due to increasing shift of the diaphragm could be an explanation for this finding. At higher PEEP levels the cranio-caudal shift of fTI decreased, a fact which could be caused by increased recruitment of caudal quadrants through higher PEEP.

The possible assessment of the distribution of tidal volumes and detection of regional differences in ventilation is a major advantage of the method and might be especially useful in settings when inhomogeneous ventilation plays a major role, such as meconium aspiration syndrome of the newborn. The method should therefore be assessed in a model of inhomogeneous lung injury and compared to imaging methods such as computer tomography in further studies.

Good agreement between VT_calc_ and VT_PNT_ was observed at PEEP = 0 cmH_2_O, even when calibration at PEEP = 3 cmH_2_O was used. Monitoring of VT after extubation, preceded by calibration with known VT during mechanical ventilation, could therefore be a possible field of application for the technique.

Another non-invasive method for the measurement of lung volume changes in neonates in the form of Optoelectronic Plethysmography has been described recently [1]. The technique was similarly accurate in measuring VT of neonates during spontaneous breathing. Whereas it has the advantage of being able to measure VT without the need for previous calibration, the setup is rather complex, which will presumably limit the applicability of the method for clinical monitoring [14]. In contrast to that, QIM with adhesive electrodes as used in the present study was simple to perform and seemed feasible for clinical conditions in neonatology. The method shares, however, the restraint of other techniques such as respiratory inductive plethysmography [15], [16], that calibration is required in order to express gas volumes as millilitres and future studies must show, how accurate volume measurements can be performed by QIM, if measuring conditions are less stable than in the present study [17].

In summary we report on a non-invasive bedside technique based on impedance measurement which allows sufficiently precise and accurate calculation of tidal volumes in ml after three-point calibration during CMV and could possibly prove as useful tool for bedside monitoring in neonatology in the future.
